# High prevalence of former elite ice hockey players requiring early hip arthroplasty surgery

**DOI:** 10.1093/jhps/hnae017

**Published:** 2024-04-18

**Authors:** Josefin Abrahamson, Ida Lindman, Pall Jónasson, Yelverton Tegner

**Affiliations:** Orthopaedic Research Unit, Sahlgrenska University Hospital, Sahlgrenska University Hospital, R-huset, Plan 7, 41380 Mölndal, Gothenburg, Sweden; Department of Orthopaedics, Institute of Clinical Sciences, Sahlgrenska Academy, University of Gothenburg, Box 100, 405 30 Gothenburg, Gothenburg, Sweden; Department of Orthopaedics, Institute of Clinical Sciences, Sahlgrenska Academy, University of Gothenburg, Box 100, 405 30 Gothenburg, Gothenburg, Sweden; Department of Orthopaedics, Institute of Clinical Sciences, Sahlgrenska Academy, University of Gothenburg, Box 100, 405 30 Gothenburg, Gothenburg, Sweden; Division of Health and Rehabilitation, Department of Health, Education and Technology, Luleå University of Technology, 971 87 Luleå, Luleå, Sweden

## Abstract

The high-impact nature of ice hockey puts the players at a higher risk of developing early hip osteoarthritis (OA). This study aims to evaluate the presence of cam morphology, early radiological findings of OA and total hip arthroplasty (THA) in former Swedish elite ice hockey players. Male elite ice hockey players in the highest league in Sweden seeking orthopedic consultation for hip and groin pain with restricted hip joint range of motion and subsequent radiographs (Antero/posterior view, Lauenstein view and/or Hip frontal view) were included. The radiographs were performed between 1988 and 2009 and retrospectively examined for the presence of cam morphology (evaluated by α-angle ≥ 60°) and hip OA (evaluated by Tönnis classification). All players were contacted between 11 and 33 years after baseline radiograph examination for follow-up investigation of the presence of subsequent THA. A total of 44 male ice hockey players were included, of which 31 had available radiographs and 39 answered the follow-up questions. Cam morphology (α-angle ≥60°) was present in 81% of the players. Seven players (18%) had received a THA with a mean age of 55.7 (SD 6.1) years at time of THA-surgery. Tönnis score at baseline radiographs were associated with THA later in life (*P* < 0.001). This study conclude that former elite Swedish ice hockey players underwent THA at a younger age than the general population. Despite confirming previous research of high prevalence of cam morphology in elite ice hockey players, no association could be established between cam morphology and the need for THA.

## INTRODUCTION

Hip and groin injuries are common in ice hockey and about half of all players in professional ice hockey report hip and groin problems over the course of one season [1, 2]. Mechanical insults to the hip joint from injuries or repeated loading (e.g. heavy lifting or sporting activities) have been reported to increase the risk of developing hip osteoarthritis (OA) [3–5]. Ice hockey players have been reported to have 2–3 times greater risk of hip OA, compared with age-matched controls [3]; however, the number of studies on ice hockey players and hip OA is limited.

Radiological hip morphology, including cam morphology and hip dysplasia, has also been suggested as risk factors for the onset of hip OA [6–10]. The radiological prevalence of cam morphology has been reported, with constantly higher prevalence in athletes, and particularly athletes participating in high-impact sports such as ice hockey, soccer, basketball and American football [11–13]. In ice hockey, goaltenders are suggested to be more exposed to intra-articular hip injuries, due to their high impacts in extreme range of motion (ROM) of the hip joint, such as the butterfly technique [14]. Furthermore, the defensemen and forwards are also suggested to place their hips in ‘at-risk’ positions, e.g. high grades of flexion, adduction and internal rotation, increasing their risk of developing cam morphology [15]. Overall, ice hockey players might therefore be at a greater risk of developing premature hip OA.

As mentioned above, studies on the development of hip OA and a subsequent total hip arthroplasty (THA) in ice hockey players are lacking. Furthermore, there is a limited number of studies on ice hockey players with cam morphology and the relationship with a subsequent THA. Increased awareness of these factors (hip OA, THA and cam morphology) in ice hockey players may alter the rationale of training programs, potentially reducing repetitive ‘at-risk’ positioning of the hips.

The aim of this study is to examine the prevalence of cam morphology, radiological findings of early OA and THA in former Swedish elite ice hockey players.

## MATERIAL AND METHODS

### Study design

Retrospective case series study design with 11–33 years follow-up.

### Study population

Inclusion criteria were Swedish male ice hockey players, active in one single team in the highest league in Sweden (the Swedish Hockey League, SHL) between 1988 and 2009 (total available Swedish male players, *n* = 95), who sought the team’s orthopedic specialist (co-author YT, chief team-physician between 1974 and 2014) with hip and groin pain, had restricted hip joint ROM upon clinical examination performed by one and the same orthopedic specialist, and were subsequently sent for radiographic examination. Exclusion criteria were female ice hockey players, and male ice hockey players with hip trauma or hip fracture visible on radiographs.

### Ethics

This study had ethical approval from the Regional Ethical Review Board in Gothenburg at the Sahlgrenska Academy, Gothenburg university, Sweden (registration number EPN 032-15), in accordance with the Helsinki Declaration.

### Data collection

In 2015, the medical records at the Hermelinen specialist medical center, Luleå, Sweden, where the team orthopedic specialist worked and documented all examinations on the ice hockey players, were identified. Search terms were ‘hockey hip’, ‘hip osteoarthritis’, ‘Idharth’ (a local specific diagnose code for sport hip arthrosis) and ICD-10 diagnosis codes used for hip and groin pain (M139, M161, M164, M167, M169, M255F, M659F, M707, M760, M796F). The medical records were retrospectively reviewed and for players who met the inclusion criteria, an evaluation of their radiographs was performed. The radiographic examinations were performed between 1988 and 2009, and then retrospectively analyzed in 2015, with evaluation for the presence of hip OA, cam morphology by one orthopedic surgeon (co-author PJ), with clinical and research experience of hip injuries in athletes since 2012.

In 2021–22, all included ice hockey players were contacted for a follow-up investigation of the presence of subsequent hip surgery. The players answered a short questionnaire including questions regarding when and why they had retired from elite ice hockey. Furthermore, they answered questions regarding if they had undergone any hip surgery, and if so, when, which hip and what type of surgery (hip arthroscopy or THA). Data on playing position were collected through a sport-specific website (https://www.eliteprospects.com/) or by the team’s material staff.

### Radiographic methods

Antero/posterior (AP) pelvic view, Lauenstein view and/or Hip frontal view were obtained. In the Hip frontal view, the leg(s) was oriented in 15° internal rotation in order to maximize the length of the femoral neck to improve its evaluability. The presence of cam morphology was evaluated by measuring the α-angle according to Nötzli et al. [16]. To define the presence of cam morphology, cut-off values of the α-angle have been varied between 50.5 and 83 [17]. In the Lisbon Agreement on Femoroacetabular Impingement Imaging from 2020, a cut-off value of 60° was, however, recommended [18]. Therefore, a cam morphology was considered present when the α-angle was 60° or higher. OA was defined according to the Tönnis classification [19]. The radiographic measurements were performed by the computer software OsiriX Lite. Intra-observer reliability was analyzed after repeating 20 of the radiograph evaluations of the α-angle 4 weeks later, showing excellent level of agreement [intraclass correlation coefficient (ICC) 0.85] [20]. Inter-observer reliability was assessed by having another experienced orthopedic surgeon re-examine the α-angle in 20 randomly selected radiographs, and indicated good level of agreement (ICC 0.73) [20].

### Statistics

Data were analyzed using SPSS (version 28.0, Armonk, NY: IBM Corp) software package. Normal distribution was tested with histograms and the Shapiro-Wilk test. Descriptive data are presented with mean (standard deviation, SD) or, in case of non-normal data distribution, median (interquartile range, Q_25_–Q_75_, IQR) for continuous variables and number (%) for categorical data. The Mann-Whitney U test was used for comparisons between the α-angle and the presence of THA. The Chi^2^ test was used for differences between categorical data. Due to the retrospective study design, no power analysis was performed *a priori*. The significance level was set at *P *< 0.05.

## RESULTS

Among the 95 Swedish ice hockey players eligible and active in the team from 1988 to 2009, 44 had consulted the team physician for hip and groin pain and were included. Thirty-one (71%) of them had available radiographs for analysis. The reasons for missing radiographs were that five players had magnetic resonance imaging (MRI), limiting the possibility of comparing data, and eight had missing data in all variables. No player was excluded due to exclusion criteria. At follow-up, 39 players (89%) answered the questionnaire in mean 22.4 (SD 4.9) years after the radiographic examination.

Table I shows demographics of the study population and Table II shows the presences of cam morphology, OA and hip surgeries (hip arthroscopy and THA). In total, 25 (81%) players had cam morphology of which 17 (in total 59%) had bilateral cam morphology. Seven players (18%) had received a THA, and two had received a THA in both hips. Mean age at time for surgery was 55.7 (SD 6.1) years. Two players were still playing ice hockey at elite level, and the rest of the players had retired from elite ice hockey at a mean age of 32.5 (SD 5.8) years. Most common reasons for retiring, as reported by the player, were: hip injury (*n* = 13) and age (*n* = 9) (Table I).

**Table I.** Demographics of the study population

**Position**, *n* (%)	
Goalkeeper	2 (4.5)
Defender	16 (36.4)
Centre/Forward	26 (59.1)
**Age at follow-up**, years, mean (SD)	54.0 (10.9)
**Age when finishing ice hockey career**, years, mean (SD)	32.5 (5.7)
**Reasons given for finishing ice hockey**, *n* (%)	
Hip injury	13 (30%)
Age	9 (20%)
Knee injury	5 (11%)
Motivation	5 (11%)
Civil career	3 (8%)
Shoulder injury	1 (2%)
Neck injury	1 (2%)
Wrist injury	1 (2%)
Missing data	4 (9%)
Still elite active	2 (5%)

SD, Standard deviation.

**Table II.** The presence of cam morphology, osteoarthritis and hip surgery

**Age at radiographic examination**, years	28.3 (8.9)
**Age at time of receiving a THA**, years	55.7 (6.1)
**α-angle**, degrees, median (IQR)	78.8 (59.9–81.9)
**Cam morphology**, number (%)	25 (81%)
**Tönnis classification, *n* (%)**	
Grade 0	20 (64%)
Grade 1	9 (29%)
Grade 2	2 (7%)
Grade 3	0 (0%)
**Hip surgery, *n* (%)**	
Arthroscopy	3 (8%)
Total hip arthroplasty	7 (18%)

Values in mean (standard deviation, SD) unless otherwise stated. IQR, interquartile range 25–75.

**Fig. 1.**
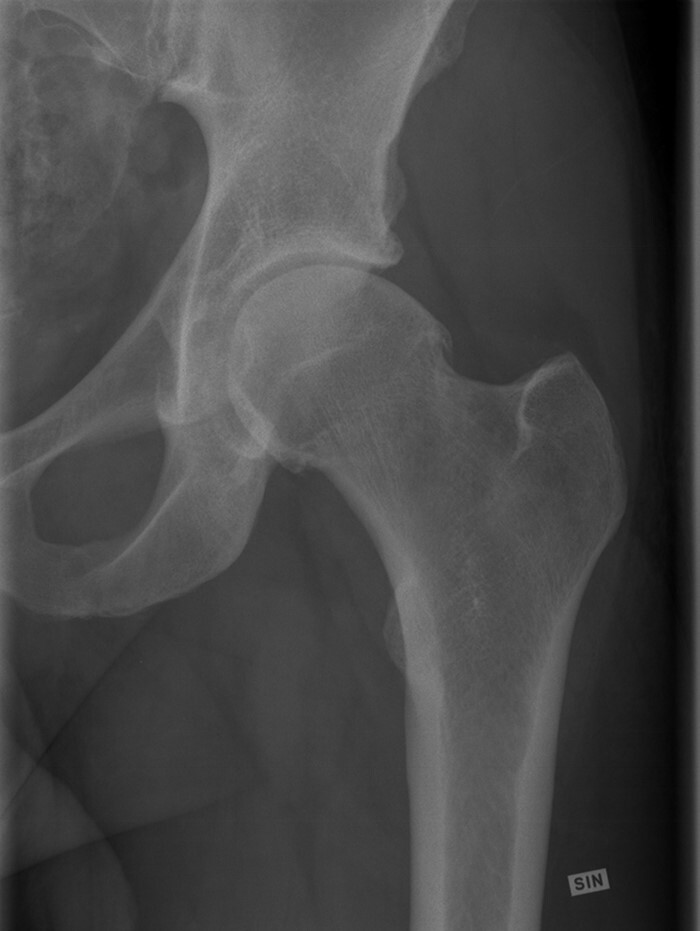
Close-up views taken from the anteroposterior pelvic radiographs showing a 35-year-old ice hockey player with cam morphology (α-angle 74°) and early osteoarthritis leading to total hip arthroplasty 15 years later.

**Fig. 2.**
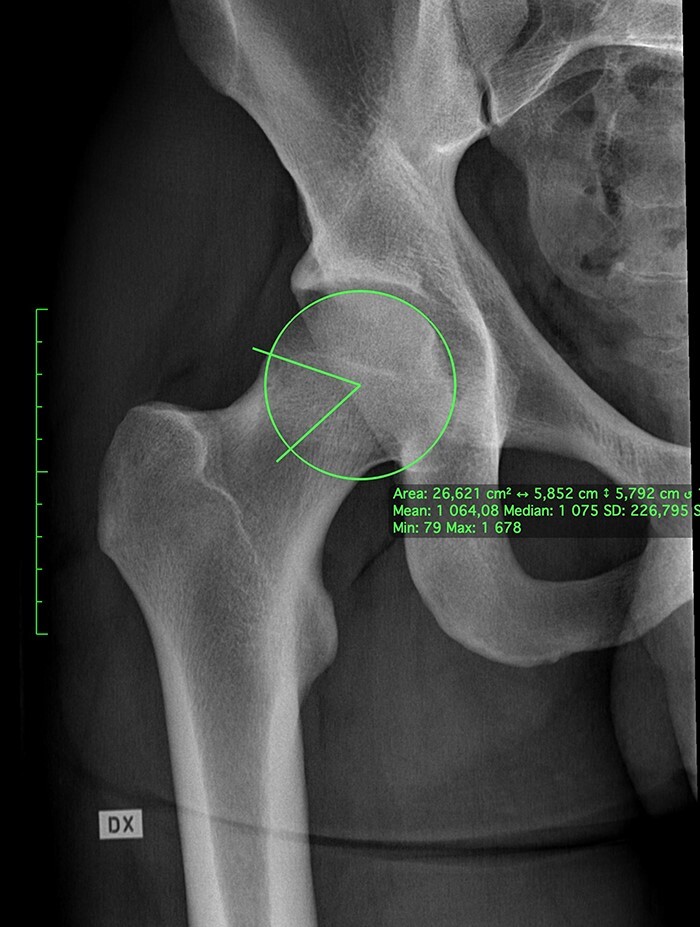
Close-up views taken from the anteroposterior pelvic radiographs showing a 21-year-old ice hockey player with cam morphology (α-angle 63°) and no early osteoarthritis.

Tönnis classification score by the time of the radiographs was associated with THA later in life (*P* < 0.001) (Fig. 1). All players with a Tönnis grade 2 at baseline and 3 out of 8 players (38%) with a Tönnis grade 1 at baseline had a THA at the follow-up. All of the seven players with THA later in life had cam morphology at the baseline radiographic examination, and the remaining 19 players (79%) with cam morphology had not received a THA (Fig. 2). No association between the α-angle or cam morphology at baseline and THA later in life was found (Table III).

**Table III.** Comparisons between baseline radiographic findings and total hip arthroplasty later in life

	THA		
	Yes	No	*P*-value
**α-angle**, median (IQR)	84.0 (80.0–88.4)	77.5 (60.3–81.8)	0.11^a^
**Cam morphology**, *n* (%)	5 (21%)	19 (79%)	0.38^b^
**Tönnis classification**, *n* (%)			**<0.001^b^**
Grade 0	0 (0%)	17 (63%)	
Grade 1	3 (11%)	5 (19%)	
Grade 2	2 (7%)	0 (0%)	

a Mann-Whitney U test.

b Chi^2^ test. THA, total hip arthroplasty. IQR, interquartile range.

## DISCUSSION

The main finding of this study was that 18% of former Swedish elite ice hockey players, who earlier in their active ice hockey careers were examined for hip and groin pain, had received a THA at a mean age of 56 years.

This study also found that 81% of the ice hockey players had cam morphology (α-angle ≥60°) at baseline radiographs. This is in line with previous studies on ice hockey players [13, 21, 22]. However, the median α-angle for the entire cohort in this study (regardless of the presence of cam deformity) was slightly higher (median 78.8°) compared with previous reports (67.7–68.8°) [22]. One reason for this discrepancy might be the fact that this study only included ice hockey players with both hip and groin pain and reduced hip ROM, while other studies include players regardless of symptoms and clinical signs.

At follow-up, 18% of the players had received a THA, of which two players in both hips. The Swedish Arthroplasty Register reported that 2.1% of the Swedish population had at least one THA at the end of 2021 [23]. Furthermore, a systematic review reported that male ice hockey players had 2–3 times greater risk of hip OA, compared with age matched controls [3]. Additionally, a recent systematic review concluded that certain sports, such as ice hockey, soccer, handball and rugby, are more frequently associated with premature knee and hip OA [24]. However, both these reviews only included two studies each on ice hockey players, indicating lack of studies in this area.

The mean age at the time of THA surgery was 56 years, which is lower than the mean age for receiving a THA in the general population in Sweden (69 years) [23]. This is in line with a previous study reporting lower age in ice hockey players being at hospital admission for hip OA compared with non-athlete controls (53.3 versus 61.2 years) [25]. All ice hockey players with THA at follow-up in the present study had cam morphology at baseline, and several studies have reported a correlation between cam morphology and early onset of hip OA [6–9]. In this study, however, the remaining 19 players who also exhibited cam morphology at baseline (i.e. 79% of all players with cam morphology) had not received a THA at the follow-up, and no association was found between the α-angle or the presence of cam morphology and THA. It must be emphasized that the sample size in this study is very small, and with a larger one an association might be statistical. Furthermore, the mean age at follow-up was 54 years, and as more years elapse, a greater number of players might have received a THA. However, other risk factors for hip OA in this study population also need to be addressed. Mechanical insults to the hip joint from repeated loading, as is inherent in the high-impact nature of ice hockey, is one. Furthermore, hip injuries, such as labral or chondral tears not caused by cam morphology, ligamentum teres tears and fractures that might have occurred between baseline and the follow-up, are others [3–5, 26].

In this cohort of ice hockey players with hip and groin problems earlier in their careers, injuries in the hip and groin were the most common reasons for finishing their elite ice hockey career. Studies have found that hip and groin injuries are common in ice hockey and that about half of all players in professional ice hockey report hip and groin problems over the course of one season [1, 2]. One conceivable aspect in this study is that all ice hockey players were examined with radiographs due to hip and groin pain and restricted hip ROM. The majority of the radiographs were performed before the 2000s, before cam morphology and femoroacetabular impingement syndrome (FAIS) had its breakthrough. Hence, the radiologist then described the cam and pincer lesions as early findings of hip OA. As no one was aware of these lesions back in the 80s, the team’s orthopedic specialist (co-author YT) therefore diagnosed it as ‘hockey hips’ due to its commonness in the team. It was not until the radiographs were retrospectively examined that it became evident that over 80% were shown with cam morphology, and would probably have been diagnosed with FAIS today. One can therefore only speculate if the ice hockey players’ careers would have been longer if they had received proper treatment for their hip problems. However, mean age for retiring from elite ice hockey was 33 years in this study, which is slightly higher than previous reports [27, 28].

There is a lack of studies investigating the prevalence of THA in ice hockey players. The long-term follow-up between 11 and 33 years after baseline examination is a strength of this study. The majority of the players were examined with AP pelvic view, which is a gold standard when assessing hip OA. Another strength is that the same orthopedic surgeon (co-author YT) performed all the clinical investigations. Furthermore, all radiological measurements were performed by the same orthopedic surgeon (co-author PJ). However, there are several limitations to this study; the small sample group may not be representative for the entire population of Swedish ice hockey players. The inclusion of only players with hip and groin pain and restricted hip ROM makes the comparability with the general population difficult to interpret and generalize. Furthermore, it is suggested that modified Dunn view should be used when assessing the hip morphology of FAIS [18], and the radiographs included in this study were AP pelvic, Dunn and Lauenstein views.

Further research on this topic is desirable, particularly longitudinal studies over the cause of many years and with larger sample sizes including players from a wider range of teams, players with and without former hip and groin problems and using more elaborate imaging.

## CONCLUSIONS

Former elite Swedish ice hockey players underwent THA at a younger age than the general population. This study also confirms previous research indicating the frequent occurrence of cam morphology in elite ice hockey players. However, within the given sample, no association could be established between cam morphology and the requirement for THA.

## Data Availability

The data from medical records (e.g. the radiographs) underlying this article cannot be shared publicly due to the Patient Safety Act (2010:659) (Patientsäkerhetslagen) in Sweden, and for the privacy of individuals that participated in this study. The data will be shared on reasonable request to the corresponding author.
